# Author’s Response to Peer Reviews of “Mass Testing With Contact Tracing Compared to Test and Trace for the Effective Suppression of COVID-19 in the United Kingdom: Systematic Review”

**DOI:** 10.2196/28744

**Published:** 2021-04-12

**Authors:** Mathew Mbwogge

**Affiliations:** 1 London School of Hygiene & Tropical Medicine London United Kingdom

**Keywords:** COVID-19, SARS-CoV-2, test and trace, universal testing, mass testing, contact tracing, infection surveillance, prevention and control, review

*This is the author’s response to peer-review reports for “Mass Testing With Contact Tracing Compared to Test and Trace for the Effective Suppression of COVID-19 in the United Kingdom: Systematic Review.”*

## Round 1 Review

Editor: We are very grateful for your valuable comments in improving this manuscript [1] so that it meets the required standard. We read every comment with much interest and addressed them accordingly. Given that our manuscript was a transfer version from a preprint server, we did not have the chance to comply with the editorial guidelines. We note that your comments, most of which were already addressed in the initially revised manuscript, have permitted us to further improve on our work. We thank you for the immense input and expertise.

1. All in-text references have now been corrected in addition to previous corrections.

2. Footnote changes were already made in the initially revised manuscript.

3. All URLs were already updated and cited in the revised manuscript.

4. We have modified the design in the title, from “rapid review” to “systematic review.”

5. The corresponding author has now been listed as recommended.

6. All major headings were already updated.

7. Subheadings were already updated as recommended.

8. We already verified that each section had at least two subsections in the initial version.

9. We have slightly modified the *Methods* subsections to mirror those in the *Results*. Each *Results* subsection, notably *Search Results*, *Methodological and Risk of Bias Assessment*, *Synthesis of Results*, and *Interstudy Variability*, has been explained in the *Methods* section under *Database Search*, *Data Quality Assessment*, *Standardized and Synthesis Metrics*, and *Heterogeneity Assessment*, respectively.

10. The reporting of *P* values has been updated with the correction of a few errors.

11. Multimedia appendices were already inserted as recommended in the previous version.

12. We have now included a statement on the study aim to wrap up the introduction.

13. A summary of findings under *Discussion* was already included.

14. Lengthy tables were already moved to the multimedia appendices section according to the guidelines.

15. The abstract was already structured according to the guidelines.

16. The results in the abstract were already fleshed up in the initially revised manuscript.

17. The references were already cleaned up in the previous version of this manuscript.

18. Percentages have been restricted to 1 decimal place and expressed in absolute values.

19. The issue of numbered headings was already corrected in the initially updated version.

20. Tables were already placed where they needed to appear in the body of the text.

21. We have cited a few more scholarly articles (some from JMIR Publications) as recommended.

22. All field codes were already removed in the previously updated manuscript.

23. Invented abbreviations were already taken care of in the previous version.

24. Not applicable to this study.

25. Not applicable to this study.

26. Tables were already modified, following the guidelines, in the previous version.

27. All figures and tables have been edited and uploaded to reflect these changes.

## Response to Reviewer H

### General Comments:

Reviewer H [2]: We are amazed by your outstanding comments and attention to detail. We cannot thank you enough for your expert knowledge and encouraging words. Your efforts in bringing this manuscript up to standard for better readership are highly applauded. We are happy to say that we agreed with all the comments and are pleased to submit a revised version.

### Specific Comments:

1. The *Research in Context* section has been sized down.

2. The section on definitions has been removed.

3. The *Data Extraction* section has been modified accordingly.

### Major Comments

1. We thank you for highlighting this. We have truly improved on the work further.

2. The review in question was cited in the *Discussion* section. However, this has now been updated.

3. Thank you very much for the valuable compliments.

4. Your comments on outcomes are very pertinent. Outcomes were defined as part of the PICO (population, intervention, comparison, and outcome of interest) statement. As a result, we have moved this to the section on eligibility criteria.

5. The section *How the Intervention Should Work* has been moved and modified as suggested. The objectives and outcomes subsection has been modified as recommended.

6. The safe nature of the mass testing and tracing program has been emphasized.

### Minor Comments

1. We have verified that a full stop has been applied to each paragraph.

2. The grammar has now been reviewed; thanks for the suggestion.

3. We have included a statement for the column regarding vote counts.
